# Supplementary material for: Time‑resolved multi-omic analysis of paclitaxel exposure in human iPSC‑derived sensory neurons unveils mechanisms of chemotherapy‑induced peripheral neuropathy
Source: Cell Death Dis. 2026 Feb 10;17(1):211. doi: 10.1038/s41419-026-08445-2 (PMC12921266; doi:10.1038/s41419-026-08445-2)
Supplement: Supplementary file 15 — Dataset 1. [file 41419_2026_8445_MOESM15_ESM.zip › CDD_Open_Data/04_RNA_Seq/Timeline_panel_plots_etc_2023-01-23_1609.zip!/Panel_Plots_etc/Evidence_Plots/48h/evidence_plot_standard contrasts_msigdb_hallmark_48h_PTX_vs_DMSO_ID5_M5892_HALLMARK_CHOLESTEROL_HOMEOSTASIS.pdf]

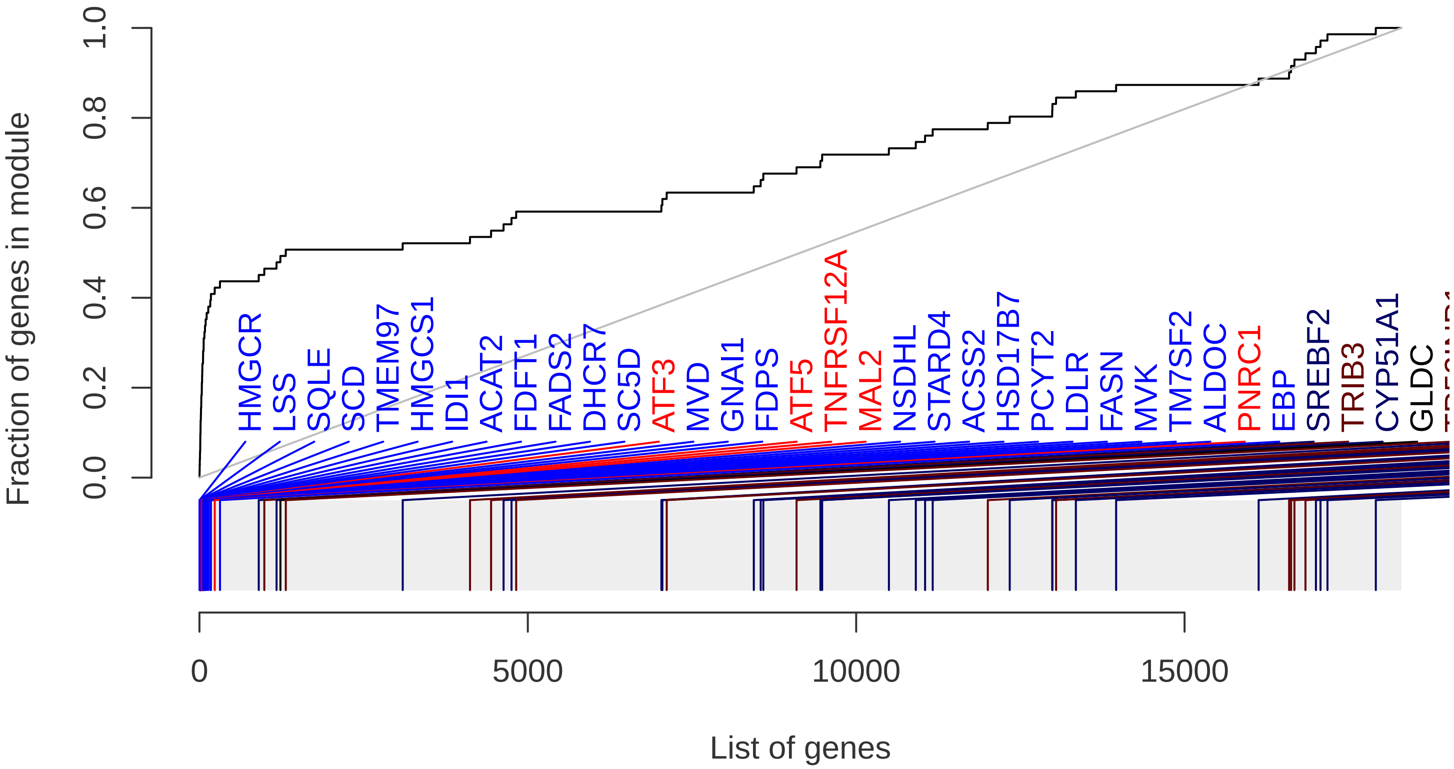
Fraction of genes in module
0.0
0.2
0.4
0.6
0.8
1.0
HMGCR
LSS
SQLE
SCD
TMEM97
HMGCS1
IDI1
ACAT2
FDFT1
FADS2
DHCR7
SC5D
ATF3
MVD
GNAI1
FDPS
ATF5
TNFRSF12A
MAL2
NSDHL
STARD4
ACSS2
HSD17B7
PCYT2
LDLR
FASN
MVK
TM7SF2
ALDOC
PNRC1
EBP
SREBF2
TRIB3
CYP51A1
GLDC
0
5000
10000
15000
List of genes
